# Digestion-Specific Acupuncture Effect on Feeding Intolerance in Critically Ill Post-Operative Oral and Hypopharyngeal Cancer Patients: A Single-Blind Randomized Control Trial

**DOI:** 10.3390/nu13062110

**Published:** 2021-06-19

**Authors:** Eyal Ben-Arie, Tzu-Hsuan Wei, Hung-Chi Chen, Tsung-Chun Huang, Wen-Chao Ho, Chiu-Ming Chang, Pei-Yu Kao, Yu-Chen Lee

**Affiliations:** 1 Graduate Institute of Acupuncture Science, China Medical University, Taichung 404, Taiwan; benarie19@gmail.com; 2 Department of Acupuncture, China Medical University Hospital, Taichung 404, Taiwan; u102030015@cmu.edu.tw (T.-H.W.); asspiderman1982@gmail.com (C.-M.C.); 3 Division of Plastic and Reconstructive Surgery, Department of Surgery, China Medical University Hospital, Taichung 404, Taiwan; d19722@mail.cmuh.org.tw (H.-C.C.); d21920@mail.cmuh.org.tw (T.-C.H.); 4 Department of Public Health, China Medical University, Taichung 404, Taiwan; wcho@mail.cmu.edu.tw; 5 Division of Thoracic Surgery, Department of Surgery, China Medical University Hospital, Taichung 404, Taiwan; 6 Surgical Intensive Care Unit, China Medical University Hospital, Taichung 404, Taiwan; 7 Chinese Medicine Research Center, China Medical University, Taichung 404, Taiwan

**Keywords:** feeding intolerance, ICU, oral cancer, hypopharyngeal cancer, acupuncture, digestion, critically ill, energy expenditure (EE), postoperative

## Abstract

Malnourishment is prevalent in patients suffering from head and neck cancer. The postoperative period is crucial in terms of nutritional support, especially after composite resection and reconstruction surgery. These patients present with a number of risk factors that aggravate feeding intolerance, including postoperative status, prolonged immobility, decreased head elevation, mechanical ventilation, and applied sedative agents. Routine management protocols for feeding intolerance include prokinetic drug use and post-pyloric tube insertion, which could be both limited and accompanied by detrimental adverse events. This single-blind clinical trial aimed to investigate the effects of acupuncture in postoperative feeding intolerance in critically ill oral and hypopharyngeal cancer patients. Twenty-eight patients were randomized into two groups: Intervention group and Control group. Interventions were administered daily over three consecutive postoperative days. The primary outcome revealed that the intervention group reached 70% and 80% of target energy expenditure (EE) significantly earlier than the control group (4.00 ± 1.22 versus 6.69 ± 3.50 days, *p* = 0.012), accompanied by higher total calorie intake within the first postoperative week (10263.62 ± 1086.11 kcals versus 8384.69 ± 2120.05 kcals, *p* = 0.004). Furthermore, the intervention group also needed less of the prokinetic drug (Metoclopramide, 20.77 ± 48.73 mg versus 68.46 ± 66.56 mg, *p* = 0.010). In conclusion, digestion-specific acupuncture facilitated reduced postoperative feeding intolerance in oral and hypopharyngeal cancer patients.

## 1. Introduction

The prevalence of general cancer patient malnourishment is currently recorded at an alarming 39–41 percent, and is a definite cause of concern [1,2]. Head and neck cancer patients have increased tendencies to display symptoms of malnourishment upon diagnosis. Throughout the course of the disease, the risk of malnourishment can increase by up to 80% [3,4]. Correspondingly, head and neck cancer patients incorporate a group of diagnostic circumstances pertaining to malignant pathologies within the upper region of the body, including the oral cavity, oropharynx, hypopharynx and larynx [5]. According to Van Deudekom et al., malnourishment is one of the particular mortality predictors in elderly patients suffering from head and neck cancer [6].

Surgical resection is the main treatment for head and neck cancer patients. After composite resection and free flap reconstruction, patients are admitted to intensive care units (ICUs) in order to allow for the close monitoring of flap and respiratory status [7]. These patients present with a number of risk factors that aggravate feeding intolerance, including postoperative status, prolonged bed ridden immobility, decreased head elevation (less than 30 °), mechanical ventilation, and the customary use of sedative agents [8]. Feeding intolerance is common in postoperative critically ill patients, and is conventionally related to poor outcomes. The indications of feeding intolerance include large residual gastric volume (RGV), aspiration, vomitus, or episodes of diarrhea [9]. The quantity of RGV in feeding intolerance varied in different studies, from 75 mL to 500 mL daily [10]. When feeding intolerance is identified, the enteral feeding volume, along with calorie intake, will not be increased in regular practice, or will be discontinued. However, both overfeeding and underfeeding are consequently related to adverse outcomes and mortality [11,12]. The patient’s optimal daily calorie intake goal, also referred to as target energy expenditure (EE), depends on both physical status and disease severity [13]. The target calories required could be obtained through indirect calorimetry, calculated by predictive equations, or by a simplistic weighted-based (25–30 kcals/kg/d) equation [13]. Zusman et al. reported that providing between 70 and 100% of measured energy expenditure was associated with improved survival [14]. The level of 70% EE is especially important for preventing mortality in patients with a high nutrition risk [12]. Current studies suggest that the specified calorie target should be achieved in 48 to 72 h after ICU admittance, and they highlight the importance of this factor in this regard [13].

Common interventions for feeding intolerance include prokinetic agent use, switching to continuous administration of enteral nutrition (EN), and post-pyloric feeding [9]. However, current interventions fail to provide complete solutions, and they do not act as a prophylactic method for feeding intolerance [15]. Acupuncture is a treatment intervention used globally for a wide variety of disorders. Its efficacy has been established over the course of 3000 years, originating in Asia and diversifying worldwide [16]. Acupuncture is considered to be a safe treatment when applied by a certified acupuncturist [17,18,19]. The significant beneficial therapeutic action of acupuncture as a safe, complementary intervention with regard to the treatment of cancer patients has been identified in an overview of 23 systematic reviews [20]. Acupuncture is commonly used in the treatment of xerostomia, pain, and toxicity after chemo-radiotherapy in head and neck cancer patients [21,22]. A prominent Cochrane meta-analysis investigating acupuncture effects in patients with symptomatic gastroparesis failed to present definite findings pertinent to this treatment as a result of a high risk of bias in the included studies, suggesting that clearly defined unbiased clinical studies could provide valuable data in this regard [23]. Two clinical studies investigated the effect of acupuncture on indigestion in ICU patients with promising results [24,25]. Another study showed that acupuncture, along with Chinese herbal medicine, can decrease the incidence of acute gastrointestinal (GI) injury in elderly patients with severe sepsis in an ICU setting [26]. However, none of the aforementioned studies investigated the treatment efficacy of acupuncture in critically ill oral and hypopharyngeal cancer patients. This highlights the need for high quality studies to provide credible evidence in this field.

In order to investigate the effect of acupuncture on postoperative feeding intolerance of critically ill oral and hypopharyngeal cancer patients, we designed a single-blind randomized control trial. We hypothesize that patients in the intervention group will reach their target EE at a faster rate when compared to the control group.

## 2. Methods

This parallel 1:1 single-blind randomized control trial was conducted from May 2019 until December 2020. The study was conducted in the surgical and burn ICUs of China Medical University Hospital in Taichung City, Taiwan. The Ethics Committee of China Medical University Hospital approved the study protocol (CMUH108-REC2–037), with the relative registration on www.clinicaltrial.gov (NCT03934294) and study protocol publication in https://journals.lww.com/md-journal/Fulltext/2019/08300/Acupuncture_effect_on_digestion_in_critically_ill.45.aspx accessed on 18 June 2021.

After the study protocol publication, we applied one modification to the inclusion criteria by removing the previous inclusion criteria: “Patients who developed feeding intolerance 2 times in the first postoperative day”. All the included patients were in the same nutrition condition where the daily calories prescribed were similar.

In addition, another modification eliminated one of the two acupuncturists from the study protocol as a result of the novel coronavirus SARS-CoV2 (COVID 19) outbreak, whereby the intention of reducing the presence of non-crucial personnel inside the ICU departments was essential. The duty of the excluded acupuncturist was to label the acupoints with a sticker prior to the acupoints needling by the second acupuncturist. The remaining acupuncturist was still blinded accordingly to the goal of the intervention and the specific set of acupoints intended for each group. A further modification was in extending the follow-up time from 3 days to 14 days. The study included two groups to be compared: An intervention group (digestion acupuncture) and a control group (non-digestion acupuncture), whereby both groups received individualized ICU care as well.

### 2.1. Inclusion Criteria

Inclusion criteria includes oral cancer and hypopharyngeal cancer patients patients aged 30–80 years. An Apache score of <20 required patients to receive EN. Only post-plastic surgery (including composite resection and flap reconstruction) patients were included.

### 2.2. Exclusion Criteria

The study excludes patients with coagulopathy (defined by prolonged prothrombin time and activated partial thromboplastin time > 4 times), thrombocytopenia, clinically unstable patients (defined as those receiving 2 inotropic agents or fraction of inspired oxygen > 70%), patient not using EN and patients with an estimated ICU stay < 3 days.

### 2.3. Randomization and Blinding

A total of 28 patients were randomized into intervention and control groups using a computer-based simple random sampling with a 1:1 ratio without stratification using the IBM SPSS ver. 22 (SPSS Inc, Chicago, IL, USA). Randomization was carried out by an individual involved in the study (EBA) who did not have any interaction with the patients. He also delivered an opaque sealed envelope to the acupuncturist. The envelope contained the patient number in the study, the acupoint′s name and the acupuncturist speculation form. The acupuncturist applied the acupuncture on the patients according to the information detailed in the envelope. Patients, study personnel and ICU staff were blind to the group allocation.

### 2.4. Interventions

When a patient was enrolled in the study, a qualified acupuncture doctor (blinded) with more than 1 year of experience applied a traditional Chinese medicine-style acupuncture intervention according to the group of allocated acupoints. The patients in both groups received interventions once a day for 3 consecutive days, totaling 3 interventions. Interventions were administered in conscious, semi-conscious or unconscious states, and throughout the post-operative sedation.

In addition to the acupuncture, the patients in both groups received individualized ICU care (including vital sign monitoring, surgical wound care, sedative medications, vasoactive medications, analgesic medications and any specific medications or lifesaving procedures prescribed by the ICU doctors as needed). Interventions started after the completion of the surgery and after the ICU admission (within day 1–3 of the postoperative days). The interventions were given by a qualified acupuncture doctor with more than 1 year of clinical experience from the acupuncture department of China medical university hospital.

### 2.5. Intervention Group (Digestion Acupuncture)

Patients in this group received traditional Chinese medicine style acupuncture with the following acupoint combination that was designed to treat indigestion related conditions according to the traditional Chinese medicine theory and recent medical research. The acupoints were: ST36 (Zusanli), ST37 (Shangjuxu), ST39 (Xiajuxu), PC6 (Neiguan), and LI4 (Hegu). Rationale for acupoints selection: In a systematic review and meta-analysis on acupuncture effect for postoperative gastroparesis syndrome, the study concluded that ST36 (Zusanli) and PC6 (Neiguan) were the most commonly used points [27]. The acupoint PC6 (Neiguan) was described to be effective for postoperative nausea and vomiting in a literature review, and the review also mentioned LI4 (Hegu) as another point that can be used for this condition [28]. The acupoint LI4 (Hegu) was also combined with ST36 (Zusanli) in a pilot study for diabetic gastroparesis and shown to reduce dyspeptic symptoms [29]. The acupoint ST37 (Shangjuxu) was used in a randomized control trial and effectively helped to reduce functional constipation in a randomized controlled trial [30]. The combination of ST36 (Zusanli), ST37 (Shangjuxu), ST39 (Xiajuxu) acupoints was shown to be an effective combination for postoperative ileus in a systematic review and meta-analysis [31]. The acupuncture included 10 needles (40 mm with 30 G needles manufactured by “Yu Kuang” company) that were applied bilaterally. Each needle was inserted into a muscle level depth and was manually manipulated to 180° on both sides in order to generate a “de chi” sensation. After 30 min, the needles were extracted by the acupuncturist. In cases where one of the patient’s limbs was wounded in the location of the acupoints, the specific wounded area was not needled. No specific instructions were given to the patients in addition to the interventions (see Supplementary Materials: Figure S1).

### 2.6. Control Group (Non-Digestion Acupuncture)

The patients in the control group received acupuncture intervention with acupoints that are not specifically indicated in the treatment of indigestion both in traditional Chinese medicine theory or in recent medical research. The acupoints were: LI 15 (Jianyu), SJ 14 (JianLiao), LU3 (Tianfu), GB35 (Yangjiao), BL 59 (Fuyang). The rationale for control acupoints selection was as follows: In the medical research, the acupoint LI 15 (Jianyu) was used in a randomized control trial for chronic neck and shoulder pain [32]. The acupoints LI 15 (Jianyu), SJ 14 (JianLiao) were defined as points that are usually used to treat shoulder pain in a cross-sectional matched case-control study [33]. The acupoint LU3 (Tianfu) was used for its effect on muscle endurance in the female and male shoulder joints [34,35]. The acupoint GB35 (Yangjiao) was used in a study that investigated the effects of aconitine via acupoint injection in rabbits [36]. For the acupoint BL 59 (Fuyang) we did not find any previous study using this point. Furthermore, we did not find any study using any of the control group acupoints for a therapeutic effect of digestion or digestion-related symptoms in the medical research literature. The acupuncture process was identical to that applied in the intervention group, but with the use of the control acupoints (see Supplementary Materials: Figure S2, Table S2).

### 2.7. Enteral Feeding Protocol and Prokinetic Drug Use

During surgery, a nasogastric (NG) tube was inserted in order to fulfill the patient’s nutritional needs. The NG tube was fixed and examined every 24 h by the ICU doctors and nurses. The NG tube was retained during the postoperative period for as long as the patient needed it. The nutritional risk and calories needed were assessed by the nutritionist before operation and ICU admission. The target EE was calculated in 2 ways as described by Yatabe et al. [13].

Predictive equations (The Harris–Benedict equations):

Men = (10 × weight in kg) + (6.25 × height in cm)−(5 × age in years) + 5

Women = (10 × weight in kg) + (6.25 × height in cm)−(5 × age in years)−161

The individual results would be multiplied by stress factors and activity factors.

Simplistic weight-based equation: 25 kcal/kg/day.

The EN was initiated on the first post-operative day as 500 mL/kcal. The amount of enteral feeding was increased gradually until the targeted calculated EE was achieved. When a patient displayed signs of feeding intolerance, 30 mg of Metoclopramide was prescribed daily, along with a change to an elemental diet and a switch to continuous administration of enteral feeding if indicated. The enteral feeding was continued, without increasing the number of calories prescribed. When there were aspirations, vomitus or diarrhea, the enteral feeding was discontinued and subsequently restarted the next day. The study nutritionists were also blind to the study group allocation.

### 2.8. Outcome Measures

Our main outcome measurement was the number of postoperative days it takes for a patient to reach his or her target EE (both equations). For secondary outcomes, we recorded the dosage of prokinetic medications used in order to treat indigestion, other treatments for indigestion, such as patients’ need for parental nutrition, the need for a post-pyloric tube, vomitus (in periods), diarrhea (in periods), and GI bleeding. Body temperature (over 38 °C), heart rate, and blood pressure before and after the interventions were monitored accordingly. We also recorded mechanical ventilation (MV) days, ICU days, days in the hospital, and mortality. The laboratory measurements focused on the variation of the serum albumin levels. We also measured the level of acupuncture doctor blinding by a speculation form conveying which group the acupuncturist thought the patients belonged to.

### 2.9. Statistical Analysis

All statistical analysis was calculated by the IBM SPSS Statistics version 22.0 (SPSS Inc., Chicago, IL, USA). The study results are displayed as a percentage (n%) for categorical data and as mean ± standard deviations for continuous data. The Mann–Whitney U and the Wilcoxon sign rank test was used in order to analyze the continuous data. The categorical data were analyzed by the Chi-square and the Fisher exact tests. The generalized linear model was used to analyse repeating measurements. The statistically significant level is defined by a *p*-value lower than 0.05. The pairwise deletion method was utilized for analyzing missing values.

## 3. Results

**Figure 1:**
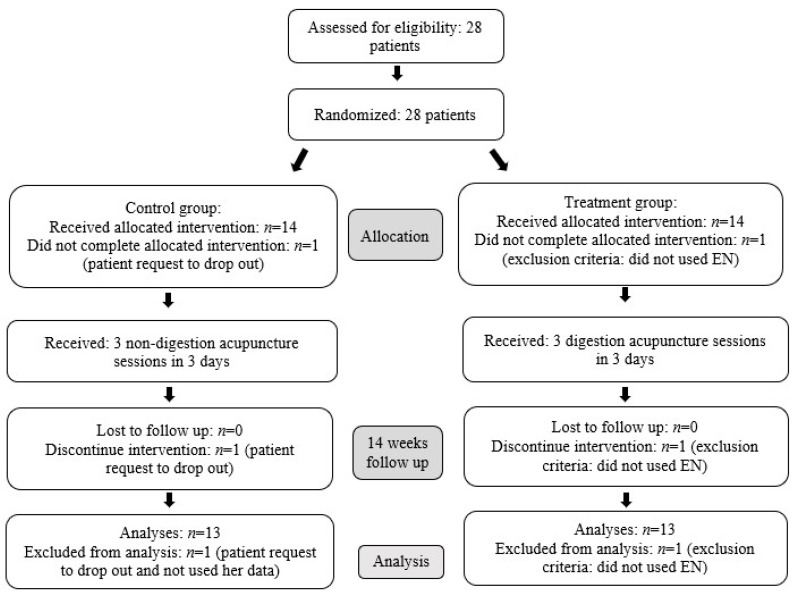
Recruitment and participant flow chart depicting information regarding enrolment, interventions, and analytic processes. Post randomization, one patient was excluded from each group and not analyzed.

A total of 28 patients were enrolled in this study from May 2019 until December 2020. After randomization of 28 patients, one patient in the intervention group was excluded as a result of the need for a free jejunal flap reconstruction resulting in failed tolerance of NG tube feeding. In the control group, one patient (female) proceeded to drop out of the study after completing two sessions of the intervention, and the subsequent data of this patient were not used as per her request. Therefore, 13 patients were analyzed in the intervention group, and 13 patients in the control group(see Figure 1). All the patients included in the study were males. The average age was 52.77 ± 6.50 in the intervention group and 55.62 ± 9.87 in the control group. One patient was a hypopharyngeal cancer patient and 12 patients were oral cancer patients in the intervention group. All 13 patients in the control group were oral cancer patients. There was no significant difference in the baseline characteristics of age, body weight, body height, Body Mass Index (BMI), target EE (calculated by the predictive equation and simplistic weight-based equation 25 kcal/kg/day), EE percent on operation day (out of the target EE), nutrition risk screening 2002, and cancer stage between the two groups (*p* > 0.05 respectively) (see Table 1).

**Figure 2:**
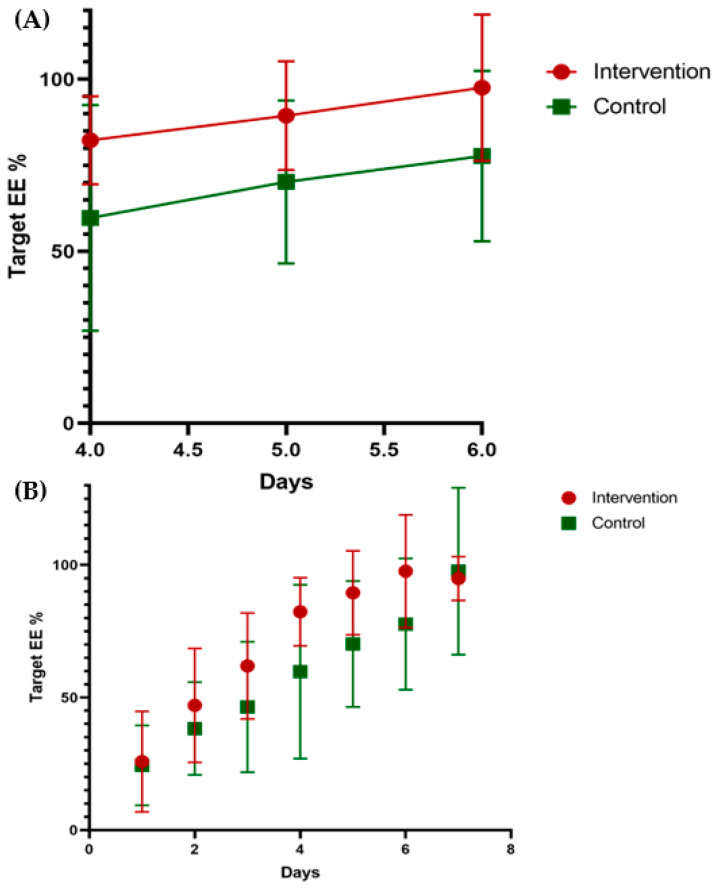
Repeat measures of EE percentages out of target EE over time. (**A**) This figure represents a visual description of a repeat measurements on days 4, 5 and 6 using a generalized linear model. Results are between groups *p* = 0.011 *, and between timelines *p* = 0.000 *. EE = energy expenditure. * refers to significant *p*-value levels lower to 0.05. (**B**) The observed tendency in reaching postoperative target EE over seven days. EE = energy expenditure.

There was a significantly earlier achievement in 70% and 80% of target EE, respectively, in both the predictive equation and the simplistic weight-based equation of the intervention group when compared to the control group (predictive equation: 3.54 ± 1.05 versus 5.62 ± 2.96 days, *p* = 0.016; simplistic weight-based equation: 3.00 ± 1.08 versus 4.92 ± 3.09 days, *p* = 0.034) (predictive equation: 4.00 ± 1.22 versus 6.69 ± 3.50 days, *p* = 0.012; simplistic weight-based equation: 3.38 ± 1.04 versus 5.38 ± 2.99 days, *p* = 0.019). There was a significantly faster time observed in reaching 100% of the target EE as calculated by the simplistic weight-based equation in the intervention group when compared to the control (4.54 ± 3.13 vs. 7.15 ± 4.12 days, *p* = 0.029) (Table 2). A repeat measurement analysis using the predictive equation showed a significant difference between the groups from the fourth to the sixth day (*p* = 0.011) (see Figure 2). The patients in the intervention group displayed a significantly increased total calorie absorption in the first week, when compared to the control group (10263.62 ± 1086.11 kcal versus 8384.69 ± 2120.05 kcal, *p* = 0.004).

For the secondary outcome measurements, patients in the intervention group were prescribed significantly less of the prokinetic drug (Metoclopramide) than the control group (20.77 ± 48.73 mg versus 68.46 ± 66.56 mg, *p* = 0.010). However, there was no significant difference in post-pyloric tube use, parental nutrition use, diarrhea, vomitus, constipation, GI bleeding, fever (more than 38°C) days, ICU days, hospital days and MV days between the two groups (*p* > 0.05 respectively) (see Table 3). Patient albumin blood levels showed a significant reduction over time in both groups from the pre-operative to the post-operative period (*p* < 0.05 within the groups, respectively). Nevertheless, there was no significance observed between the two groups in the postoperative measurements in serum albumin levels (*p* > 0.05) (see Table 4). Heart rate and blood pressure measurements showed no significant change within and between the groups before and after acupuncture on the first to the third days of measurements (*p* > 0.05 respectively). Data on incidents of nausea were not available for data collection due to the postoperative sedation (patients did not report). Concerning the study blinding, the blind acupuncturist group speculation was correct in 91.6% in both the intervention group and the control group interventions (see Supplementary Materials: Table S1). No adverse events were observed in this study.

## 4. Discussion

This is the first study investigating the effect of acupuncture on postoperative feeding intolerance in oral and hypopharyngeal cancer patients within an ICU setting. The intervention group reached the level of 70% and 80% of the target EE significantly earlier than the control group in both EE equations, and the intervention group also reached the level of 100% of the target EE significantly earlier than the control group when calculated by the simplistic weight-based equation (4.54 ± 3.13 vs. 7.15 ± 4.12 days, *p* = 0.029). For the secondary outcome, patients in the intervention group were prescribed less prokinetic drugs than the control group patients (20.8 ± 48.7 mg versus 68.5 ± 66.6 mg, *p =* 0.010). The intervention group also observed a shorter ICU stay (7.92 ± 7.52 days vs. 8.23 ± 4.30 days, *p* = 0.169), a shorter hospital stay (18.92 ± 5.38 versus 22.77 ± 10.22 days, *p =* 0.418), and fewer MV days (5.00 ± 3.34 versus 6.54 ± 3.93 days, *p =* 0.223). Although these findings were not considered statistically significant, nonetheless they serve to provide valuable insight.

Malnutrition in head and neck cancer patients is common, with a prevalence ranging from 20 to 80% [4,37]. The possible morbidities associated with malnutrition are increased risk of infection, delayed wound healing, impaired function of cardiac and respiratory systems, muscle weakness, depression, post-operative complications, reduced response to chemotherapy and radiotherapy, and an increased mortality rate [38]. For head and neck cancer patients, a few medical alternatives were performed to improve the patients’ nutrition status. In Paleri et al.’s review on gastrostomy placement in head and neck cancer patients, the prophylactic use of gastrostomy did not benefit patients’ nutrition status and was related to negative impact in quality of life [39]. Moreover, Hausmann et al. reported that the wound infection rate of gastrostomy could be as high as 40% [40]. In addition, preoperative early nutrition administration was not related to an improved post-operative nutrition status [41]. For the modification of diet formula, glutamine supplement reduced the incidence of mucositis in chemotherapy and arginine was related to less infection and a shorter hospital stay [42,43]. However, none of the diet modifications proved to be related to ameliorated enteral intake and gastrointestinal symptoms [42,43,44]. In the present study, due to the complexity of reconstructive surgery, all the participants were admitted to an ICU and 88.46% (23 over 26 patients) of them experienced sedation for the medical needs. Narcotics were also prescribed for pain control and the patients were kept bed-ridden for the wound care. The nutrition treatment in critically ill patients in the acute stage (such as a postoperative stage) will require a gradual increase in the percent of the target EE, as in steps of 25% daily increase [45]. However, it is common for patients to reach their target EE after a period of more than 10 days [46]. Compher et al.’s study on 2853 mixed ICU patients from 202 ICUs showed that patients only reached 62% of their target EE by day 4 in the ICU [47]. Auiwattanakul et al. conducted a study on 1,686 surgical ICU patients in Thailand and reported that patients reached their calorie goal after 7–10 days [48]. Another study on 170 surgical ICU patients in Thailand found that less than 40% of the patients reached 60% of their target calories absorption by the fourth day in the ICU [49]. The tendency of developing feeding intolerance and decreased calorie absorption as a result of ICU stays is a common observation that may impair the patient outcome. Our study found that patients in the intervention group managed to reach 80% of their target EE (calculated by predictive equation) on the fourth day postoperatively while the control group only reached 60% of their target EE, which is compatible to other studies. The earlier intestinal recovery of patients in the intervention group also facilitates the explanation of the fact that patients in this group had a significantly higher actual mean number of weekly calories delivered compared to the control group. The positive impact on reaching the target EE in the intervention group also benefits the general outcome of the patient presented as a shorter ICU stay (7.92 ± 7.52 versus 8.23 ± 4.30 days), less MV time (5.00 ± 3.34 versus 6.54 ± 3.93 days), and shorter hospital stay (18.92 ± 5.38 versus 22.77 ± 10.22 days), although these changes are not statistically significant.

The current treatment for indigestion in critically ill patients is the use of prokinetic drugs such as intravenous metoclopramide or its combination with erythromycin. However, these drugs are associated with detrimental side effects such as QT prolongation, diarrhea, and tardive dyskinesia [50]. In this study, we found that patients in the intervention group were prescribed less prokinetic drugs than the control group (Metoclopramide, 20.77 ± 48.73 mg versus 68.46 ± 66.56 mg, *p* = 0.010). The drug erythromycin was not routinely used in the ICU where we conducted the study. This significant reduction of prokinetic drug use indirectly demonstrates the beneficial effect of the digestion-specific acupuncture points used in the intervention group. The above-mentioned findings take into consideration that all the included patients were male patients. This can be rationalized according to the fact that the study population of oral cancer patients and hypopharyngeal cancer are predominately males [51,52,53]. Furthermore, the male prevalence in oral and hypopharyngeal cancers is even more profound in the Taiwanese population [54,55]. Thus, the interpretation of the results should reflect this detail accordingly, and the treatment efficacy should not immediately be generalized to influence all gender populations. In oral cancer patients, age differences can affect the kind of treatment that patients will receive, as elderly patients might not receive adjuvant chemo-radiation therapy [56]. The average age of oral cancer patients in Asia is 51–55 years old [57]. The patients’ mean age in this clinical trial was in that range (52.77 ± 6.50 in the intervention group and 55.62 ± 9.87 in the control group). Likewise, the findings should not be generalized for other age groups. The patients’ severity level might be considered to be lower when compared to other ICU patients, since the patients required ICU care to primarily provide immediate postoperative and sedative care and therefore would not be expected to need a long ICU stay. According to Chen et al., the need for ICU observation in oral cancer patients after a flap surgery is debatable [58]. Therefore, our findings should not be generalized to reflect on other critically ill patients. Since the included patients are postoperative patients, and the operation location restricts oral feedings, the findings of our study should also not be generalized to reflect other kinds of postoperative patients that still have oral feeding abilities. Nausea is a common undesirable postoperative symptom that occurs in up to 30% of patients [59]. The fact that this study has missing data on incidents of nausea can serve as a significant limitation. The incidents of nausea were one of the study outcome measurements in the protocol stage, and the study findings did not include data on incidents of nausea since the patients did not report the feeling of nausea to the ICU nurses. The patients were under postoperative sedation, or not in a fully conscious state, which prevented the reporting of the incidents of nausea to the nurses in most of the study time points. Another possible explanation for the missing data is the location of the surgery. The surgery was applied on or in close proximity to the oral cavity, a circumstance that may temporarily impair the patients’ ability to talk and report any discomfort to ICU nurses.

Our study directs focus at the efficacy of acupuncture treatment in oral and hypopharyngeal cancer patients over a short period of time. Future studies should be conducted in order to investigate the efficacy of acupuncture over long-term treatment interventions, whereby the number of sessions is increased. Finally, it is also important to not overly generalize the findings, as the study only included a small sample size.

The mechanism associated with the acupuncture effect on the digestive system can be partially explained by the way specific acupuncture points can influence gastric and duodenal motility in rats. According to Noguchi et al., acupuncture-related increased motility is achieved through the supra-spinal reflex that activates vagal nerve fibers, while decreased motility is associated with a spinal reflex that activates sympathetic efferent nerve fibers [60]. Qin et al. showed that the acupuncture point ST37 (Shangjuxu, used in the present study) was associated with increased jejunal motility through activation of C fibers and M2 receptors in rats [61]. Tatewaki et al. showed that the acupuncture point ST36 (Zusanli, used in the present study), can regulate gastric contractions in rats with increasing motility when motility is low, and decreasing motility in hypermotility via vagal efferent and opioid pathways [62]. The acupuncture point PC 6 (Neiguan, used in the present study) also had an ability to increase gastric motility in rats by increasing peripheral efferent vagus nerve fiber discharge. When the vagus nerve was disabled, the effect was majorly reduced [63]. In a clinical trial conducted by Jin et al., acupuncture showed remarkable effectiveness in treating functional dyspepsia symptoms, improving mental status, and improving the overall quality of life. The study explains its effect via increased frequency and propagation speed of gastric slow waves, as well as increased serum gastrin secretion. The study used the acupuncture points ST 36 (Zusanli), as the main point and PC 6 (Neiguan) as a secondary point among other points [64]. A literature review conducted by Li et al. further explained the effect of acupuncture on the regulation of GI function, whereby the review showed that acupuncture can both increase and decrease the gastric peristalsis depending on the patient’s needs. The review explains the mechanism of acupuncture on GI motility through the following pathways: transient receptor potential vanilloid-1 (TRPV1) receptor, N-methyl-D-aspartate receptors, parasympathetic efferent pathway, oxytocin expression, brain-gut peptide, vagal pathway and opioid pathway [65].

In recent years, there have been a handful of studies investigating the effect of acupuncture on indigestion in critically ill patients. Pfab et al. showed a constant reduction in gastric residual volume in the acupoint stimulation group compared to a metoclopramide group in traumatic critically ill brain injury and stroke patients [24]. Meng et al. investigated the effect of electro-acupuncture (EA) for critically ill septic patients, and found a reduction in the score of intestinal dysfunction in the EA group [66]. Wang et al. also found an improvement in GI dysfunction following EA combined with Chinese herbal medicine compared with ICU routine care in critically ill patients after abdominal surgery [67]. A non-randomized observational multicenter study investigated the effect of acupuncture along with Chinese herbal medicine treatment vs. routine ICU treatment (control) in 296 severe sepsis patients for the prevention of acute gastrointestinal injury. The study found a significant decrease in the levels of D-lactic acid, diamine oxidase, endotoxin, gastrin, and intra-abdominal pressure, and a significant increase in the levels of motilin in the acupuncture group compared to the control [26]. Our study strongly contributes reliable evidence of positive acupuncture treatment effects in critically ill patients with regard to indigestion.

The task of designing a double-blind study in acupuncture research is often a serious challenge. Similar to invasive interventions such as surgery, it is difficult to blind the doctor performing the acupuncture. In our study, we designed an attempt to blind the acupuncture doctors from the intervention goal, which was to improve digestion and prevent feeding intolerance. The acupuncture doctors were presented with one of two point selection options, and they had to guess if they treated the intervention or the control group. Our results showed accurate determinations of groups in more than 90% of the cases. Therefore, this study was labeled a single blind trial in order to avoid study bias. A possible way to perform double-blind acupuncture studies is to use placebo-sham acupuncture needles as shown in other studies [68,69,70]. However, the use of placebo needles limits the needling depth and the ability to manipulate the needles.

## 5. Conclusions

In conclusion, digestion-specific acupuncture can decrease the number of days patients need to reach key levels of target EE, increase the total weekly calorie intake and reduce the use of prokinetic drugs. These findings suggest that the use of specific acupuncture can significantly reduce feeding intolerance in critically ill postoperative oral and hypopharyngeal cancer patients. The functional implementation of acupuncture treatment renders it a safe therapeutic intervention for oral and hypopharyngeal cancer patients in the postoperative period. However, the interpretation of the results should take into consideration limitations in sample size, patients’ gender, age, and disease severity. High quality studies are required to provide more evidence of the functional role of this treatment with regard to feeding intolerance, clinical application and safety. Future research is needed in order to verify our findings prior to extensive applications.

## 6. Limitations

In this study there were serval limitations. The study sample size serves as a limitation, since only 28 patients were enrolled in this study. For target EE calculation, although the indirect calorimetry was recommended, it was not available in our ICU, and is not available in many other ICUs. In future studies, measuring the target EE using an indirect calorimetry would provide more accurate and individual tailored target EE results. Data on incidents of nausea were missing from the study result due to the patient condition and this serves as a limitation. The acupuncture doctor blinding did not prove effective since accurate deductions of specific groups ensued, serving as a limitation. Patient blinding was not measured but was assumed, as the patients were not in a fully conscious state, and both groups received acupuncture interventions. The fact that patients’ blinding levels were not measured could possibly serve as a limitation. The study only included male patients, as oral and hypopharyngeal cancers is less common in females. Hence, further studies including female patients and larger sample sizes should be conducted in the future.

## Figures and Tables

**Table 1:** Baseline characteristics.

		Intervention Group (*n* = 13)	Control Group (*n* = 13)	*p*-value
Age		52.77 ± 6.50	55.62 ± 9.87	0.479
Gender (M)		13 (100%)	13 (100%)	1.00
Weight		63.39 ± 12.99	68.95 ± 15.52	0.579
Height		167.44 ± 5.36	170.50 ± 5.32	0.081
BMI		22.54 ± 4.34	23.62 ± 4.69	0.687
No. of patients with BMI				
<18.5		3(23%)	1(7.7%)	0.511
18.5–20.5		1(7.7%)	3(23%)	0.511
>20.5		9(69.2%)	9(69.2%)	1.000
Target EE (kcal)	Predictive equation	2031.23 ± 146.93	2029.62 ± 195.52	0.880
	Simplistic weight-based equation	1584.81 ± 324.64	1723.85 ± 387.89	0.579
BEE (kcal)		1418.23 ± 217.86	1490.85 ± 279.97	0.687
Nutrition risk screening 2002:	Low risk	0 (0%)	0 (0%)	1.00
	Medium risk	3 (23.1%)	0 (0%)	0.336
	High risk	10 (76.9%)	13(100%)	0.336
Cancer stage:	Stage I	2 (15.4%)	2 (15.4%)	1.00
	Stage II	3 (23.1%)	1 (7.7%)	0.569
	Stage III	1 (7.7%)	4 (30.8%)	0.252
	Stage IV	6 (46.2%)	3 (23.1%)	0.376
	Recurrent cancer status	1(7.7%)	1(7.7%)	1.00
Types of surgery:	Primary tumor resection with reconstruction	8 (61.5%)	11(84.6%)	0.336
	Functional or cosmetic reconstruction	5(38.5%)	2(15.4%)	0.336
Previous free flaps		2.08 ± 1.80	1.46 ± 0.78	0.579
Previous cancer-related surgeries		3.23 ± 2.98	1.62 ± 0.87	0.243

M = male, EE = energy expenditure, BEE = basal energy expenditure, BMI = Body Mass Index. Data presented as mean ± standard deviation, or as number of patients and percent (%).

**Table 2:** Target EE (in kcal) and the duration required to achieve it in both groups.

	Intervention Group (*n* = 13)	Control Group (*n* = 13)	*p*-Value
Target EE by predictive equation(kcal)	2031.23 ± 146.93	2029.62 ± 195.52	0.880
70% of target EE(days)	3.54 ± 1.05	5.62 ± 2.96	0.016 *
80% of target EE	4.00 ± 1.22	6.69 ± 3.50	0.012 *
100% of target EE	8.31 ± 4.03	8.62 ± 3.84	0.545
Target EE by simplistic weight-based equation(kcal)	1584.81 ± 324.64	1723.85 ± 387.89	0.579
70% of target EE(days)	3.00 ± 1.08	4.92 ± 3.09	0.034 *
80% of target EE	3.38 ± 1.04	5.38 ± 2.99	0.019 *
100% of target EE	4.54 ± 3.13	7.15 ± 4.12	0.029 *
Total Calories intake for first week	10263.62 ± 1086.11	8384.69 ± 2120.05	0.004 *

EE = energy expenditure. Data are presented as mean ± standard deviation. * Refers to significant *p*-value levels of lower to 0.05.

**Table 3:** Secondary outcomes revealed significantly less use of the prokinetics drug in the intervention group.

	Intervention Group (*n* = 13)	Control Group (*n* = 13)	*p*-Value
Prokinetic drug use (Metoclopramide, mg)	20.77 ± 48.73	68.46 ± 66.56	0.010 *
Post-pyloric tube use	1(7.7%)	0	0.762
Parental nutrition use	0	1(7.7%)	0.762
Diarrhea (days) Constipation (days)	1.46 ± 1.76 5.92 ± 2.56	2.23 ± 2.17 4.62 ± 2.40	0.418 0.311
Fever days (>38 °C)	2.62 ± 2.47	3.00 ± 2.83	0.545
ICU days	7.92 ± 7.52	8.23 ± 4.30	0.169
Hospital days	18.92 ± 5.38	22.77 ± 10.22	0.418
MV days	5.00 ± 3.34	6.54 ± 3.93	0.223
Mortality	0	0	1.00

ICU = intensive care unit, MV = mechanical ventilation. Data are presented as mean ± standard deviation. * Refers to significant *p*-value levels lower to 0.05.

**Table 4:** The albumin level dropped significantly in both groups after operation.

		Intervention Group (*n* = 13)	Control Group (*n* = 13)	*p*-Value (Between Groups)
Albumin	Before OP	3.75 ± 0.71(*n* = 8)	3.84 ± 0.86(*n* = 8)	0.798
	After OP	3.18 ± 0.39(*n* = 13)	2.93 ± 0.43(*n* = 12)	0.186
*p*-value (within groups)		0.042 *	0.036 *	

There was no difference between groups. IV Albumin = intravenous albumin, OP = operation. Data are presented as mean ± standard deviation, and number of patients (n). * Refers to significant *p*-value levels lower to 0.05.

## Data Availability

The datasets used and/or analyzed during the current study are available from the corresponding author on reasonable request.

## Supplementary Materials

The following are available online at https://www.mdpi.com/article/10.3390/nu13062110/s1, Figure S1: Acupuncture points for treatment group, Figure S2: Acupuncture points for control group, Table S1: Acupuncturist group speculation, Table S2: STRICTA 2010 checklist.

## Abbreviations

EN	Enteral Nutrition
PN	Parenteral Nutrition
ICU	Intensive Care Unit
GI	Gastrointestinal
EE	Energy Expenditure
COVID-19	Novel Coronavirus SARS-CoV2
IV	Intravenous
NG	Nasogastric
MV	Mechanical Ventilation
BMI	Body Mass Index
ESICM	European Society of Intensive Care Medicine
OP	Operation
HR	Heart Rate
BP	Blood Pressure
Reccurent	Recurrent of oral cancer
RGV	Residual Gastric Volume
