# Supplementary material for: Digestion-Specific Acupuncture Effect on Feeding Intolerance in Critically Ill Post-Operative Oral and Hypopharyngeal Cancer Patients: A Single-Blind Randomized Control Trial
Source: Nutrients. 2021 Jun 19;13(6):2110. doi: 10.3390/nu13062110 (PMC8234819; doi:10.3390/nu13062110)
Supplement: Supplementary file 1 [file nutrients-13-02110-s001.zip › nutrients-1244818-supplementary.pdf]

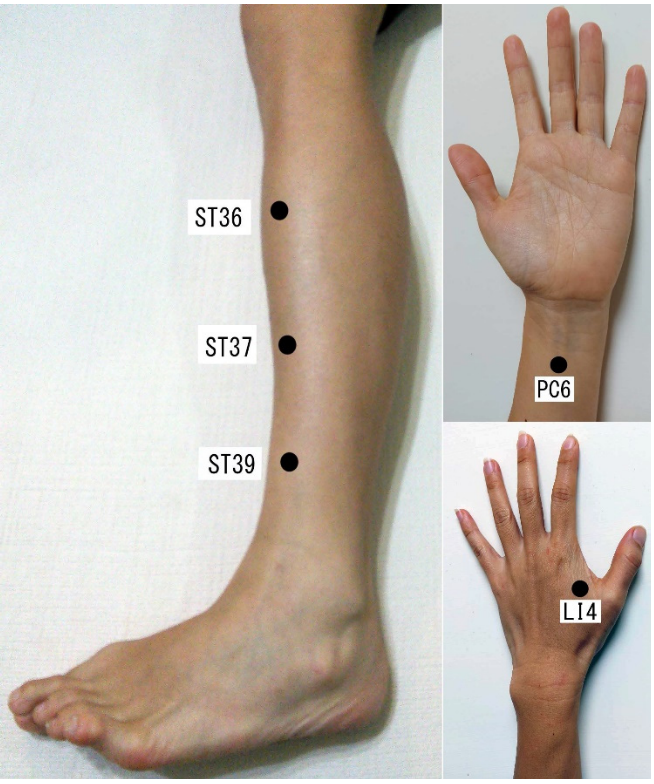

Figure S1. Acupuncture points for treatment group. Figure Legend: The Figure represent acupoint that are indicated to treat indigestion and indigestion related symptoms. The acupoints were: ST36 (Zusanli), ST37 (Shangjuxu), ST39 (Xiajuxu), PC6 (Neiguan), and LI4 (Hegu).

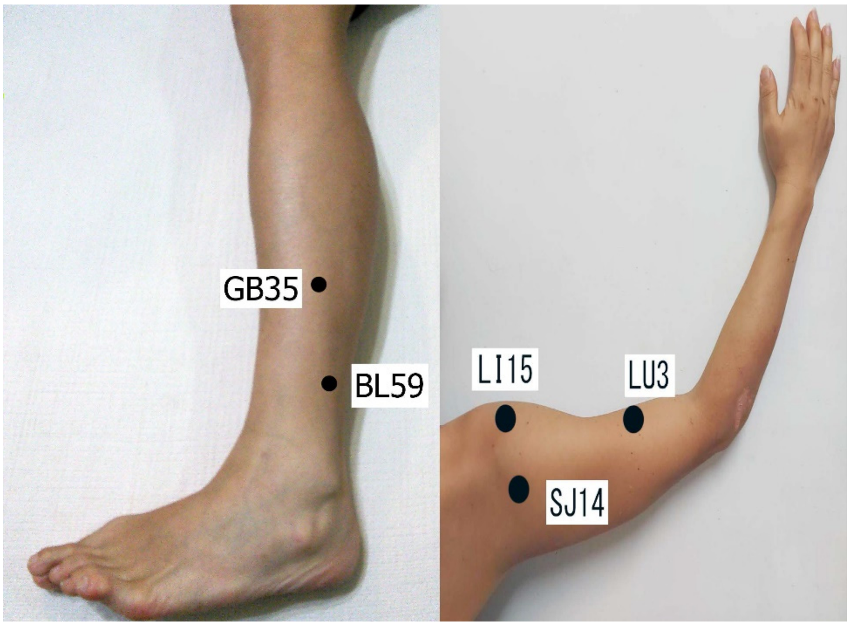

Figure S2. Acupuncture points for control group. Figure Legend: The Figure represent acupoint that are not indicated to treat indigestion or indigestion related symptoms. The acupoints were: LI 15 (Jianyu), SJ 14 (JianLiao), LU3 (Tianfu), GB35 (Yangjiao), BL 59 (Fuyang).

Table S1. Acupuncturist group speculation.

	Intervention group	Control group	P-value
Guess correct	12 (91.6%)	12 (91.6%)	1.000
Guess wrong	1 (8.3%)	1 (8.3%)	1.000

Supplementary Table 1: The table describes the percentage of acupuncturist correct and incorrect guess for each intervention. Data are presented as N and percentage values, * refers to significant P-value levels lower to 0.05.

Table S2. STRICTA 2010 checklist.

Item	Detail	Page
**1. Acupuncture rationale**	1a) Style of acupuncture (e.g. Traditional Chinese Medicine, Japanese, Korean, Western medical, Five Element, ear acupuncture, etc)	4
	1b) Reasoning for treatment provided, based on historical context, literature sources, and/or consensus methods, with references where appropriate	4,10, published protocol
	1c) Extent to which treatment was varied	4
**2. Details of needling**	2a) Number of needle insertions per subject per session (mean and range where relevant)	4
	2b) Names (or location if no standard name) of points used (uni/bilateral)	4
	2c) Depth of insertion, based on a specified unit of measurement, or on a particular tissue level	4
	2d) Response sought (e.g. *de qi* or muscle twitch response)	4
	2e) Needle stimulation (e.g. manual, electrical)	4
	2f) Needle retention time	4
	2g) Needle type (diameter, length, and manufacturer or material)	4
**3. Treatment regimen**	3a) Number of treatment sessions	4
	3b) Frequency and duration of treatment sessions	4
**4. Other components of treatment**	4a) Details of other interventions administered to the acupuncture group (e.g. moxibustion, cupping, herbs, exercises, lifestyle advice)	4
	4b) Setting and context of treatment, including instructions to practitioners, and information and explanations to patients	4
**5. Practitioner background**	5) Description of participating acupuncturists (qualification or professional affiliation, years in acupuncture practice, other relevant experience)	4
**6. Control or comparator interventions**	6a) Rationale for the control or comparator in the context of the research question, with sources that justify this choice	5
	6b) Precise description of the control or comparator. If sham acupuncture or any other type of acupuncture-like control is used, provide details as for Items 1 to 3 above.	5

Note: This checklist, which should be read in conjunction with the explanations of the STRICTA items provided in the main text, is designed to replace CONSORT 2010's item 5 when reporting an acupuncture trial.

Citation: MacPherson H, Altman DG, Hammerschlag R, Youping L, Taixiang W, White A, Moher D; STRICTA Revision Group. Revised STandards for Reporting Interventions in Clinical Trials of Acupuncture (STRICTA): extending the CONSORT statement. PLoS Med. 2010 Jun 8;7(6):e1000261
